# The effects of PSA kinetics on the outcome of hypofractionated salvage radiotherapy for biochemical recurrence of prostate cancer after prostatectomy

**DOI:** 10.1093/jrr/rraa074

**Published:** 2020-09-05

**Authors:** Hitoshi Ishikawa, Keiko Higuchi, Takuya Kaminuma, Yutaka Takezawa, Yoshitaka Saito, Toru Etsunaga, Kazushi Maruo, Hidemasa Kawamura, Nobuteru Kubo, Takashi Nakano, Mikio Kobayashi

**Affiliations:** Department of Radiation Oncology, Isesaki Municipal Hospital, Tsunatorimoto 12-1, Isesaki, 372-0802 Gunma, Japan; Hospital of the National Institute of Radiological Sciences, National Institutes for Quantum and Radiological Sciences and Technology, Anagawa 4-9-7, Inage, 263-8555 Chiba, Japan; Department of Radiation Oncology, Isesaki Municipal Hospital, Tsunatorimoto 12-1, Isesaki, 372-0802 Gunma, Japan; Department of Radiation Oncology, Gunma University Graduate School of Medicine, Showa 3-39-22, Maebashi, 371-8511 Gunma, Japan; Department of Urology, Isesaki Municipal Hospital, Tsunatorimoto 12-1, Isesaki, 372-0802 Gunma, Japan; Department of Urology, Isesaki Municipal Hospital, Tsunatorimoto 12-1, Isesaki, 372-0802 Gunma, Japan; Department of Urology, Isesaki Municipal Hospital, Tsunatorimoto 12-1, Isesaki, 372-0802 Gunma, Japan; Department of Biostatistics, Faculty of Medicine, University of Tsukuba, Tennodai 1-1-1, Tsukuba, 305-8575 Ibaraki, Japan; Department of Radiation Oncology, Gunma University Graduate School of Medicine, Showa 3-39-22, Maebashi, 371-8511 Gunma, Japan; Department of Radiation Oncology, Gunma University Graduate School of Medicine, Showa 3-39-22, Maebashi, 371-8511 Gunma, Japan; Hospital of the National Institute of Radiological Sciences, National Institutes for Quantum and Radiological Sciences and Technology, Anagawa 4-9-7, Inage, 263-8555 Chiba, Japan; Department of Radiation Oncology, Gunma University Graduate School of Medicine, Showa 3-39-22, Maebashi, 371-8511 Gunma, Japan; Department of Urology, Isesaki Municipal Hospital, Tsunatorimoto 12-1, Isesaki, 372-0802 Gunma, Japan

**Keywords:** salvage radiotherapy, prostate-specific antigen, prostate cancer, hypofractionated radiotherapy, prostate-specific antigen doubling time, biochemical recurrence

## Abstract

The feasibility and efficacy of hypofractionated salvage radiotherapy (HS-RT) for prostate cancer (PC) with biochemical recurrence (BR) after prostatectomy, and the usefulness of prostate-specific antigen (PSA) kinetics as a predictor of BR, were evaluated in 38 patients who received HS-RT without androgen deprivation therapy between May 2009 and January 2017. Their median age, PSA level and PSA doubling time (PSA-DT) at the start of HS-RT were 68 (53–74) years, 0.28 (0.20–0.79) ng/ml and 7.7 (2.3–38.5) months, respectively. A total dose of 60 Gy in 20 fractions (three times a week) was three-dimensionally delivered to the prostate bed. After a median follow-up of 62 (30–100) months, 19 (50%) patients developed a second BR after HS-RT, but only 1 patient died before the last follow-up. The 5-year overall survival and BR-free survival rates were 97.1 and 47.4%, respectively. Late grade 2 gastrointestinal and genitourinary morbidities were observed in 0 and 5 (13%) patients, respectively. The PSA level as well as pathological T-stage and surgical margin status were regarded as significant predictive factors for a second BR by multivariate analysis. BR developed within 6 months after HS-RT in 11 (85%) of 13 patients with a PSA-DT < 10 months compared with 1 (17%) of 6 with a PSA-DT ≥ 10 months (median time to BR: 3 vs 14 months, *P* < 0.05). Despite the small number of patients, our HS-RT protocol seems feasible, and PSA kinetics may be useful for predicting the risk of BR and determining the appropriate follow-up schedule.

## INTRODUCTION

As in Western countries, the incidence of prostate cancer (PC) in Japan is increasing. Vital Statistics of Japan, published by the Ministry of Health, Labour and Welfare, estimated that ~100 000 new PC patients were diagnosed with, and more than 12 000 patients died from PC in 2018 [1]. Radical prostatectomy (RP) with lymph node dissection is the standard treatment for localized PC, as well as radiotherapy (RT). However, many studies have reported 5-year rates of biochemical recurrence (BR) after RP of 10–20%, even in patients with low- to intermediate-risk PC, and the rate of a positive surgical margin (PSM) based on pathological examination is also 10–20% [2–4]. Although recent advances in surgical technique such as laparoscopic RP and robot-assisted laparoscopic prostatectomy have resulted in less invasive approaches, especially in terms of the volume of blood loss and duration of hospitalization [5], neither BR nor PSM rates have improved [6].

RT is effective as salvage therapy for PC patients with BR and/or PSM after RP. Trock *et al*. reported a better cancer-specific survival rate in patients who received salvage RT (S-RT) than in those without S-RT [7]. A systematic review of the treatment outcomes after S-RT in patients with BR showed a sigmoid dose–response curve for estimating BR-free survival (bRFS) rates [8], and the serum level of prostate-specific antigen (PSA) at the start of S-RT affected bRFS as did pathological features after RP, such as the Gleason score (GS), extracapsular involvement and surgical margin status [8–10]. Furthermore, the PSA doubling time (PSA-DT) is another risk factor for BR after S-RT [7, 8, 11, 12], although no definitive cutoff value has been established. The Japanese Radiation Oncology Study Group conducted a multi-institutional retrospective study of S-RT for PC patients with BR and found the PSA level before S-RT to be a predictive marker of BR, but PSA kinetics were not evaluated because of a lack of data during and after S-RT [13].

Radiobiologically, hypofractionated RT can improve oncologic outcomes in terms of toxicity and tumor control, because the α/β ratio is estimated to be lower in PC cells than in organs at risk such as the rectum and bladder [14, 15]. Recently, many clinical studies of primary PC have confirmed the feasibility and efficacy of hypofractionated RT when moderate hypofractionation at a fractional dose of 2.5–4.0 Gy was applied [16, 17]. On the other hand, studies of hypofractionated S-RT (HS-RT) are still limited, especially in Japan. In a retrospective study by the Japanese Radiation Oncology Study Group in 2015, 180 (97%) of 186 patients received S-RT at a total dose of ≤70 Gy using a conventional fractionation schedule [13].

In our institute, S-RT and adjuvant RT using a hypofractionation schedule at a daily dose of 3 Gy were first used for PC patients after RP in 2003. Hence, the feasibility of HS-RT for PC patients with BR after prostatectomy and the impact of PSA kinetics before and after S-RT on clinical outcomes, especially bRFS, were evaluated in the present study.

**Table 1:** Patient characteristics

Factors	Number of patients (%)
Age (years)	Median: 68, range: 53–76
≤69	25 (66%)
≥70	13 (34%)
Pathological T stage	
pT2a/b	2 (5%)
pT2c	23 (61%)
pT3a	8 (21%)
pT3b	5 (13%)
Gleason score	
5/6	4 (10%)
7	22 (58%)
8	3 (8%)
9	9 (24%)
Lymphatic invasion	
No	14 (37%)
Yes	24 (63%)
Venous invasion	
No	34 (90%)
Yes	4 (10%)
Neural invasion	
No	14 (37%)
Yes	24 (63%)
Margin status	
Negative	28 (74%)
Positive	10 (26%)
PSA level before RP (ng/ml)	Median: 9.86; range: 4.1–60.16
≤9.99	19 (50%)
≥10.00	19 (50%)
PSA level before HS-RT (ng/ml)	Median: 0.28; range: 0.20–0.79
≤0.450	29 (76%)
≥0.451	9 (24%)
PSA-DT before HS-RT (months)	Median: 7.7; range: 2.3–38.5
≤11.9	27 (71%)
≥12.0	11 (29%)
Time from RP to HS-RT (months)	Median: 40; range: 5–119
≤35	16 (42%)
≥36	22 (58%)
Type of RP	
ORP	20 (52%)
LRP	12 (32%)
RARP	6 (16%)

## MATERIALS AND METHODS

### Patients

Between May 2009 and January 2017, 51 patients were treated with HS-RT for BR after RP. Among them, 13 (25%) patients were excluded from the present study because androgen deprivation therapy (ADT) was combined with the HS-RT (*n* = 9) or the PSA level did not decrease below 0.2 ng/ml after RP (*n* = 4). Thus, the remaining 38 patients treated with HS-RT without ADT were evaluated, and their patient and tumor characteristics are summarized in Table 1. The median age was 68 years, ranging from 53 to 76 years. Twenty (52%), 12 (32%) and 6 (16%) patients had undergone open radical prostatectomy (ORP), laparoscopic radical prostatectomy (LRP) and robot-assisted prostatectomy (RARP), respectively. Histopathological examination revealed a pathological T (pT)-stage of pT2a, pT2c, pT3a and pT3b, according to the 7th edition of the UICC TNM staging system, in 2 (5%), 23 (61%), 8 (21%) and 5 (13%) tumors, respectively, and a GS of ≤ 6, 7 and ≥8 in 4 (10%), 22 (58%) and 12 (32%) tumors, respectively. A PSM was observed in 10 (26%) tumors. The median PSA level and PSA-DT before S-RT were 0.28 (0.20–0.79) ng/ml and 7.7 (2.3–38.5) months, respectively. All 38 patients received abdominal CT as a pre-HS-RT screening, but 6 (16%) and 11 (29%) did not undergo MRI and bone scintigraphy, respectively.

### Treatments

HS-RT was performed at a total dose of 60 Gy in 20 fractions with a fractional dose of 3 Gy and 3 fractions per week (Monday, Wednesday and Friday). The schedule was determined according to our previous reports of definitive 3D conformal RT combined with or without high-dose-rate brachytherapy for PC [18, 19]. The dose distribution in each treatment plan was calculated using Ecripse ver. 7.3^®^ (Varian Medical Systems, Palo Alto, CA, USA) until March 2013 and Ecripse ver. 11.00^®^ (Varian Medical Systems) thereafter. Based on the CT images taken at 2.0 or 2.5 mm intervals, the clinical target volume in each patient was defined as the prostate bed, by referencing MRI images before RP. The planning target volume was determined by three-dimensionally adding a 10 mm margin to the clinical target volume in all directions, except for a 5 mm margin in the posterior direction. Appropriate leaf margins were designed by checking each treatment plan to cover the planning target volume using six portals, but the initial six treatments were performed using four portals. In principal, the posterior margins for two opposed lateral beams were reduced to decrease the rectal volume irradiated at high RT doses after 51 Gy in 17 fractions. HS-RT was performed using 6 MV X-rays delivered by the Clinac-2100C linear accelerator (Varian Medical Systems) until March 2013 and then 10 MV X-rays delivered by the Clinac iX system (Varian Medical Systems) thereafter.

**Fig. 1.**
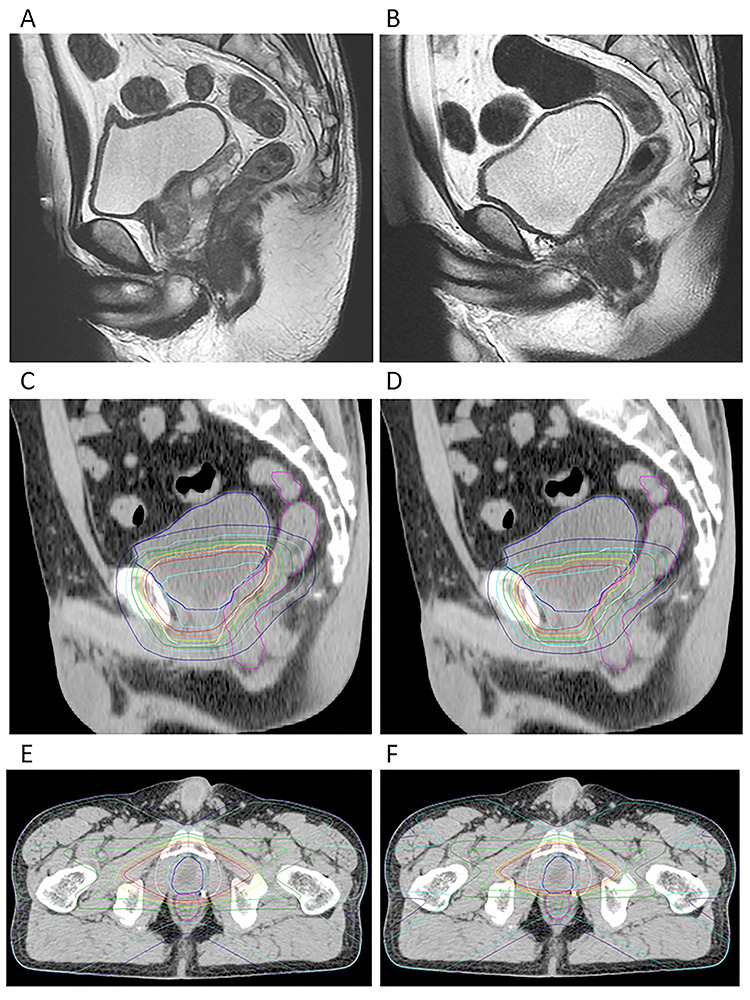
Representative dose distribution of hypofractionated salvage radiotherapy. MRI images before (**A**) and after (**B**) radical prostatectomy. Dose distributions: sagittal views of the initial (**C**) and boost (**D**) plans and axial views of the initial (**E**) and boost (**F**) plans (isodose lines: 95% in red; 90% in orange, 80% in yellow, 70% in yellow–green, 50% in green, 30% in light-blue, and 10% in blue).

Referring to our results from hypofractionated RT for localized prostate cancer [18], we attempted not to exceed 20 and 30% for percentages of the entire rectal volumes receiving 90 (54 Gy) and 80% (48 Gy) of the prescribed irradiation dose (60 Gy), respectively, although strict dose constrains for the rectum using dose–volume histogram analysis were not used in the present study. In addition, maximum radiation doses to femoral head and small intestine were set to be 50 Gy. A representative dose distribution is shown in Fig. 1.

Each treatment was performed with the patient in the supine position without respiratory gating. For patient position verification, 30 (79%) patients treated since March 2013 received HS-RT under image guidance using cone-beam CT referring to the border of the rectum and bladder after bone matching BrainLAB ExacTrac X-ray 6D stereotactic localization system (BrainLAB AG, Feldkirchen, Germany) because of renewal of the RT system at our hospital. For 8 patients, who had been treated before February 2013, unreceived image-guided HS-RT, the radiation isocenter was verified using computed radiography by 6 MV X-ray at the first session of HR-ST and reconfirmed if necessary.

### Follow-up and evaluation of the prostate-specific antigen level

Follow-up examinations were performed at 3-month intervals during the first year and at 3–6-month intervals thereafter. If the PSA level decreased below 0.2 ng/ml after HS-RT and then increased again to ≥0.2 ng/ml, the date of BR was defined as the date that the PSA level exceeded 0.2 ng/ml. If the PSA level did not decrease below 0.2 ng/ml after HS-RT, the date of the first PSA measurement after HS-RT was determined as the date of BR, as defined previously [13, 20]. If recurrence was visible by diagnostic imaging, these cases were judged as clinical recurrence. Complications were assessed in each patient after HS-RT according to the National Cancer Institute’s Common Terminology Criteria for Adverse Events, version 5 [21].

### Statistical analysis

Overall survival (OS) was defined from the date of starting HS-RT to the date of death or the last follow-up. bRFS or clinical recurrence-free survival (cRFS) was defined from the date of starting HS-RT to the date of BR or clinical recurrence, respectively, death, or the last follow-up. The cumulative rate of each survival type was calculated using the Kaplan–Meier method and is expressed as a percentage with 95% confidence intervals (CIs). The factors predicting bRFS were examined by univariable and multivariable Cox proportional hazard models, where the variable selection was conducted using the stepwise method with the inclusion and exclusion criterion of *P*-value = 0.2 in the multivariable model. Although the covariates in the Cox models were basically dichotomized, the multivariate model using continuous covariates was also conducted as the sensitivity analysis. Demographic and clinical parameters were compared using the chi-square test or Fisher’s exact test for categorical variables and Student’s *t* test for continuous variables. Differences were considered significant at a *P*-value < 0.05. All data were analyzed using SAS version 9.4 (SAS Institute Inc., Gary, NC, USA). The present study was approved by the institutional ethical committee (No. 2017–24).

## RESULTS

### Treatment outcomes

**Fig. 2.**
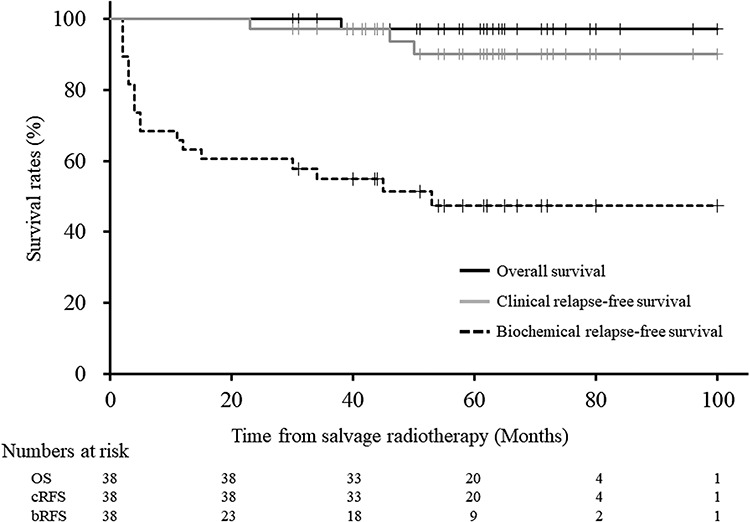
Kaplan–Meier curves for overall, clinical recurrence-free and biochemical recurrence-free survival rates after hypofractionated salvage radiotherapy in patients with biochemical recurrence of prostate cancer after radical prostatectomy.

The last follow-up examination was performed in December 2019, and all but one patient, who died of tumor progression at 38 months after HS-RT, were still alive and periodically followed up. The median follow-up time of the survivors was 62 (range 30–100) months. BR after HS-RT developed in 19 (50%) patients, and the median interval from the start of HS-RT to BR was 4 (range 2–53) months. Among these 19 patients, 3 (16%) experienced clinical recurrence at the bone and/or lymph nodes. The 5-year rates of OS, bRFS and cRFS were 97.1% (82.3–99.6), 47.4% (31.4–63.9) and 90.2% (73.3–96.8), respectively (Fig. 2).

The acute and late toxicities that developed are summarized in Table 2, and dose–volume histogram data of the rectum in the present study are shown in Table 3. No grade 3 genitourinary (GU) or gastrointestinal (GI) toxicities occurred after HS-RT. Grade 2 acute GU and GI toxicities developed in 1 and 2 patients, respectively. Regarding late toxicity, no grade 2 GI toxicity was observed, but late grade 2 GU toxicities developed in 5 (13%) patients. The 5-year cumulative rates of grade 2 GU and GI toxicities were 12.0 (4.5–19.5) and 0%, respectively.

### Prognostic factors

Table 4 summarizes the univariate and multivariate analyses results and the 5-year bRFS rates according to the prognostic factors evaluated in the present study. According to the multivariate analysis, pT-stage, PSM and PSA level before HS-RT were independent prognostic factors (*P* < 0.05).

Table 5 shows a comparison of the background characteristics between the patients with BR and those without BR. The characteristics defined as prognostic factors were confounding each other. For example, BR after HS-RT was not observed in any of the 6 tumors with PSM among the 25 patients with T2 disease but did develop in 9 (47%) of the 19 tumors without PSM. Similarly, among the 13 patients with T3 disease, BR was observed in 8 (89%) of the 9 tumors with a PSM and in 2 (50%) of the 4 without a PSM.

**Table 2:** Acute and late toxicities after hypofractionated salvage radiotherapy

	Grade 0	Grade 1	Grade 2	Grade 3
Acute				
Genitourinary	21 (55%)	16 (42%)	1 (3%)	0 (0%)
Gastrointestinal	32 (84%)	4 (11%)	2 (5%)	0 (0%)
Late				
Genitourinary	26 (68%)	7 (18%)	5 (13%)	0 (0%)
			Urinary frequency: 2	
			Hematuria: 1	
			Incontinence: 1	
			Dysuria: 1	
Gastrointestinal	36 (95%)	2 (5%)	0 (0%)	0 (0%)

**Table 3:** Percentages of rectal volume receiving 10–90% of the prescribed doses in 10% increments

	All	4 portals	6 portals	*P*-value
	(*n* = 38)	(*n* = 6)	(*n* = 32)	(4 vs 6 portals)
V90	9.9 ± 5.5	18.1 ± 7.1	8.4 ± 5.2	0.11
V80	14.6 ± 6.9	23.9 ± 8.0	12.9 ± 6.6	0.08
V70	19.9 ± 8.6	28.3 ± 8.6	18.4 ± 8.6	0.12
V60	27.8 ± 10.9	33.5 ± 10.4	26.8 ± 11.1	0.29
V50	34.5 ± 13.4	44.6 ± 21.2	32.6 ± 12.3	0.14
V40	40.3 ± 14.4	50.7 ± 22.5	38.4 ± 13.3	0.15
V30	47.0 ± 14.3	55.1 ± 21.2	45.5 ± 13.5	0.24
V20	52.4 ± 14.5	59.6 ± 19.6	51.1 ± 14.1	0.27
V10	60.3 ± 15.1	66.7 ± 16.8	59.1 ± 15.1	0.27

**Table 4:** Predictive factors for biochemical recurrence after HS-RT

Univariate analysis		
Factors	5 year bRFS (95% CI)	*P*-value
Age (years)		
≤69 (*n* = 25)	39.9% (19.8–59.4)	0.361
≥70 (*n* = 13)	61.5% (30.8–81.8)	
pT stage		
T2 (*n* = 25)	60.4% (36.8–77.6)	0.020
pT3 (*n* = 13)	20.5% (3.8–46.3)	
Gleason score		
≤7 (*n* = 26)	62.2% (39.1–78.6)	0.004
≥8 (*n* = 12)	12.5% (0.9–39.9)	
Lymphatic or venous invasion		
No (*n* = 13)	71.4% (40.6–88.2)	0.098
Yes (*n* = 25)	32.5% (13.6–53.0)	
Neural invasion		
No (*n* = 14)	68.8% (35.7–87.3)	0.063
Yes (*n* = 24)	34.9% (16.2–54.5)	
Margin status		
Negative (*n* = 28)	35.9% (17.8–54.4)	0.068
Positive (*n* = 10)	80.0% (40.9–94.6)	
PSA level before RP (ng/ml)		
≤9.99	46.1% (20.2–68.6)	0.721
≥10.0	47.4% (24.4–67.3)	
PSA level before HS-RT (ng/ml)		
≤0.450 (*n* = 29)	56.1% (35.0–72.7)	0.034
≥0.451 (*n* = 9)	22.2% (3.4–51.3)	
PSA-DT (months)		
≤11.9 (*n* = 27)	40.0% (21.7–57.8)	0.058
≥12.0 (*n* = 11)	68.2% (29.7–88.6)	
Time from RP to HS-RT (months)		
≤35 (*n* = 16)	50.0% (24.5–71.0)	0.801
≥36 (*n* = 22)	46.0% (23.1–66.2)	
Type of RP		
Open (*n* = 20)	48.0% (27.1–69.6)	
LRP/RARP (*n* = 18)	48.6% (26.7–71.0)	0.991
**Multivariate analysis**		
**Factors**	**HR (95% CI)**	***P*-value**
Age (years)		
≤69	Reference	
≥70	0.40 (0.13–1.21)	0.105
pT stage		
pT2	Reference	
pT3	3.88 (1.46–10.30)	0.006
Margin status		
Negative	Reference	
Positive	0.19 (0.04–0.87)	0.032
PSA level before HS-RT (ng/ml)		
≤0.450	Reference	
≥0.451	3.38 (1.19–9.56)	0.022

HR = hazard ratio

**Table 5:** Background characteristics of the patients with vs without biochemical recurrence

Factors	No recurrence	Recurrence	*P*-value
	(*n* = 19)	(*n* = 19)	
Age pT stage	68 (53–74)	66 (53–76)	0.828
pT2 (*n* = 25)	16 (84%)	9 (12%)	0.022
pT3 (*n* = 13)	3 (23%)	10 (77%)	
Gleason score			
≤7 (*n* = 26)	17 (65%)	9 (35%)	0.013
≥8 (*n* = 12)	2 (17%)	10 (83%)	
Lymphatic or venous invasion			
No (*n* = 13)	10 (77%)	3 (23%)	0.019
Yes (*n* = 25)	9 (36%)	16 (64%)	
Neural invasion			
No (*n* = 14)	10 (79%)	4 (21%)	0.046
Yes (*n* = 24)	9 (38%)	15 (62%)	
Margin status			
Negative (*n* = 28)	17 (61%)	11 (39%)	0.035
Positive (*n* = 10)	2 (20%)	8 (80%)	

### Prostate-specific antigen kinetics as a predictor of biochemical recurrence

**Fig. 3.**
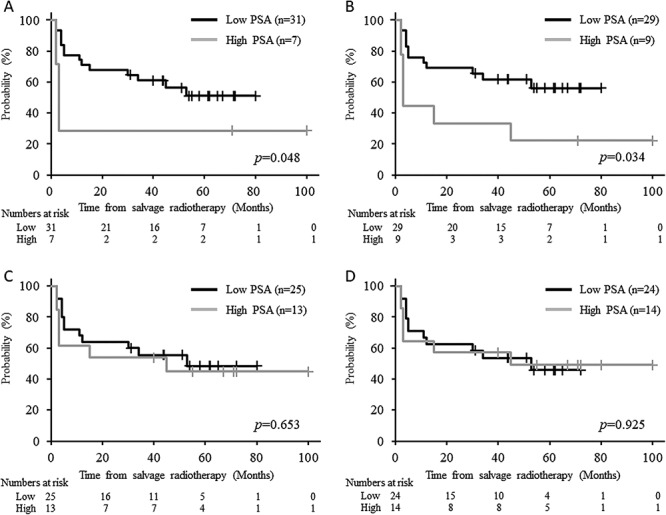
Kaplan–Meier curves for biochemical recurrence-free survival according to different cutoff prostate-specific antigen (PSA) levels before hypofractionated salvage radiotherapy. Cutoff PSA levels: 0.5 ng/ml (**A**), 0.45 ng/ml (**B**), 0.35 ng/ml (**C**) and 0.30 ng/ml (**D**). No patient had a PSA level of 0.35–0.45 ng/ml.

The bRFS curves according to the cut-off PSA level before HS-RT are shown in Fig. 3. When the cut-off PSA level was set to 0.45 ng/ml, the 5-year bRFS rates in the low and high PSA groups were 56.1 (35.0–72.7) and 22.2% (3.4–51.3), respectively (*P* = 0.034, Fig. 3). The PSA levels before HS-RT in the patients with BR and those without BR were 0.39 ± 0.18 and 0.34 ± 0.14 ng/ml, respectively, but the difference was not significant (*P* = 0.45).

**Fig. 4.**
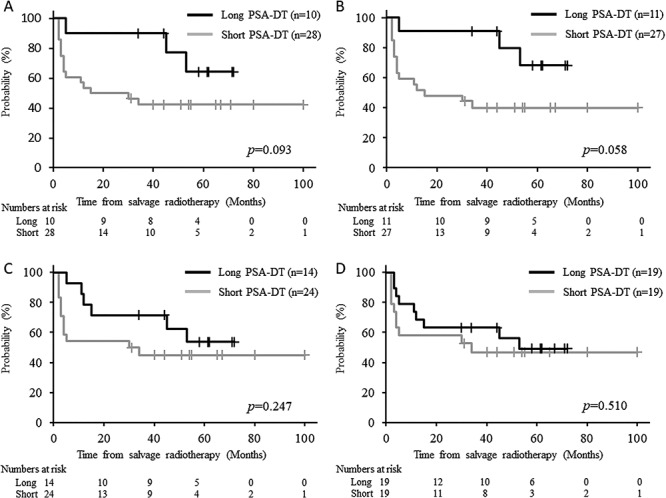
Kaplan–Meier curves of biochemical recurrence-free survival according to different cutoff prostate-specific antigen doubling times (PSA-DTs) before hypofractionated salvage radiotherapy. Cutoff PSA-DT: 14 months (**A**), 12 months (**B**), 10 months (**C**) and 8 months (**D**).

Figure 4 shows the bRFS curves according to the PSA-DT. When the cut-off PSA-DT was 12 months, the 5-year bRFS rates in the short and long PSA-DT groups were 40.0 (21.7–57.8) and 68.2% (29.7–88.6), respectively, and the difference tended to be significant (*P* = 0.058) (Fig. 4). The PSA-DTs before HS-RT in the patients with BR and those without BR were 9.8 ± 8.6 and 11.7 ± 10.9 months, respectively, but the difference was not significant (*P* = 0.55). The PSA level before HS-RT was not correlated with any background characteristic of the patients, whereas PSA-DT before HS-RT was strongly correlated with the pT-stage, GS and neural invasion (Table 6).

**Table 6:** Background characteristics according to the PSA level and PSA-DT before HS-RT

	PSA level (ng/ml)		*P*-value	PSA-DT (months)		*P*-value
	≤0.450	>0.450		<12	≥12	
Factors	(*n* = 29)	(*n* = 9)		(*n* = 27)	(*n* = 11)	
Age (median) pT stage	67 (53–74)	68 (55–76)	N.S.^a^	66 (53–76)	68 (55–74)	N.S.
pT2 (*n* = 25)	19 (76%)	6 (24%)	N.S.	14 (56%)	11 (44%)	0.013
pT3 (*n* = 13)	10 (77%)	3 (23%)		13 (100%)	0 (0%)	
Gleason score						
≤7 (*n* = 26)	22 (85%)	4 (15%)	N.S.	15 (58%)	11 (42%)	0.022
≥8 (*n* = 12)	7 (58%)	5 (42%)		12 (100%)	0 (0%)	
Lymphatic or venous invasion						
No (*n* = 13)	11 (85%)	2 (15%)	N.S.	8 (72%)	5 (28%)	N.S.
Yes (*n* = 25)	18 (87%)	7 (13%)		19 (76%)	6 (23%)	
Neural invasion						
No (*n* = 14)	11 (79%)	3 (21%)	N.S.	6 (43%)	8 (57%)	0.011
Yes (*n* = 24)	18 (75%)	6 (25%)		21 (88%)	3 (12%)	
Margin status						
Negative (*n* = 28)	20 (71%)	8 (29%)	N.S.	20 (71%)	8 (29%)	N.S.
Positive (*n* = 10)	9 (90%)	1 (10%)		7 (70%)	3 (30%)	

^a^N.S. = not significant.

**Fig. 5.**
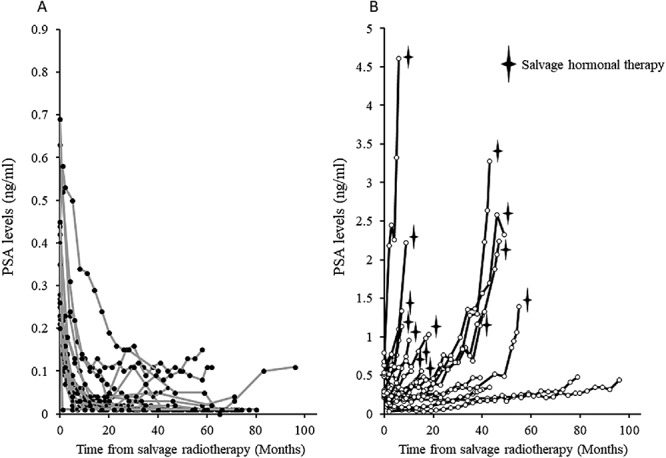
Change in the prostate-specific antigen level after hypofractionated salvage radiotherapy in patients with (**A**) or without (**B**) biochemical recurrence.

Figure 5 shows the change in the PSA level after HS-RT in the patients with BR and those without BR. The PSA level within 6 months after HS-RT showed a > 50% reduction compared with the level at the start of HS-RT in 18 (95%) of the 19 patients without BR. On the other hand, the PSA level in all but 1 (95%) patient with BR (*n* = 19) did not decrease by > 50% compared with the baseline level until 6 months after HS-RT. Of the 19 patients with BR, the PSA level in 12 (63%) never decreased below 0.2 ng/ml after HS-RT, and 15 (79%) developed BR within 12 months after HS-RT according to our criteria. Furthermore, 11 of 13 (85%) BRs observed among 24 tumors with a PSA-DT < 10 months developed within 6 months after HS-RT, but 5 (83%) of 6 BRs among the remaining 14 tumors with PSA-DT ≥ 10 months developed >6 months after HS-RT; the median times of BR development after HS-RT in the short and long PSA-DT groups were 3 (range 2–34) and 14 (range 5–53) months, respectively (*P* < 0.05).

### DISCUSSION

In the present study, a total dose of 60 Gy in 20 fractions with a fractional dose of 3 Gy was used for HS-RT. When the α/β values of the prostate, rectum and bladder were set to 2.0, 3.0 and 5.0, their biologically effective doses using our dose-fractionation schedule were estimated to be 150, 120 and 96 Gy, respectively, corresponding to 75, 72 and 69 Gy equivalent doses in 2.0 Gy fractions (EQD2) according to the linear quadratic model [22, 23].

Several important points were observed in this study. First, our HS-RT regimen seems feasible, because there were no late grade 2 or more severe GI toxicities over a median follow-up of >5 years. Ishiyama *et al*. reported a rate of grade 2 or more severe GI toxicities of 9% after HS-RT (total dose of 70 Gy in 32 fractions, fractional dose of ~2.2 Gy) among patients treated in the USA [24], and the rectal EQD2 dose in their study was almost the same as that in our study. One probable reason for the relatively lower incidence of GI toxicity in the present study is our fractionation schedule, in which three fractions a week were delivered to avoid late adverse events in the rectum, based on a previous study [18]. The rectal EQD2 of our HS-RT delivered over a week corresponded to <10 Gy in 5 fractions, as calculated by the linear quadratic model.

Conversely, it was unclear whether high-dose irradiation using hypofractionation helped prevent BR in our PC patients compared with standard-dose irradiation using conventional fractionation. In this study, BR was observed in 47% of the patients, and the 5-year bRFS and cRFS rates were 47.4 and 90.2%, respectively, over a median follow-up of 62 months. These rates were slightly better than those reported recently by other Japanese studies (5-year bRFS rates: 38–41% after S-RT at a total dose of 65–70 Gy using 1.8–2.0 Gy daily fractionation) [24–26], but we cannot directly compare these results because of difference in dose fraction, use of rectal balloon, and techniques of positioning and beam delivery. On the other hand, a multi-institutional Japanese study of S-RT in 186 patients without ADT showed 5-year bRFS and cRFS rates of 50.1 and 90.1%, respectively [13]. Although the biologically effective dose was higher in the present study than in that multi-institutional study, in which almost all patients received 60–70 Gy via conventional fractionation, similar outcomes were obtained in both studies. However, differences in the patients’ backgrounds between the studies could not be determined because of a lack of detailed information in their study.

The risk factors for a second recurrence of PC after S-RT have been determined by previous studies [7, 8, 11–13, 20, 25, 26]. In a single-institutional retrospective study, Kinoshita *et al*. reported a 5-year bRFS rate of 42.1% after S-RT at a total dose of 66 Gy in 33 fractions [27]. In their study, a high GS, negative surgical margin and high PSA nadir after RP were significant predictors of a worse bRFS. Our study also identified a high GS and negative surgical margin as significant predictors of a worse bRFS, in addition to the pT-stage and pre-HS-RT PSA level and PSA-DT; we could not evaluate the PSA nadir level because of missing data in a few patients. The percentages of patients with a GS ≥ 8 and a negative surgical margin in the present study were 32% (12 of 38 patients) and 74 (28 of 38 patients), respectively, whereas the corresponding rates in the study by Kinoshita *et al*. were 24% (12 of 49 patients) and 43% (21 of 49 patients) [27]. Moreover, we excluded patients who received HS-RT combined with ADT whereas 14% of their patients received S-RT combined with ADT. Thus, our study included a larger population of patients at high risk of a second BR after S-RT; nevertheless, favorable results were obtained. Although the optimal total irradiation dose for S-RT is unclear, a systematic review showed a remarkable dose–response correlation in terms of bRFS after S-RT for recurrent PC [8]. Hence, dose escalation in hypofractionated RT seems to be a promising method for definitive S-RT.

Our study indicates that evaluation of PSA kinetics before and after HS-RT is an important tool for not only predicting bRFS but also determining the follow-up schedule for each patient. The 5-year bRFS rate was significantly worse in patients with a PSA level >0.45 ng/ml at the start of HS-RT than in those with lower PSA levels (22.2 vs 56.1%, *P* = 0.034). A multivariate analysis revealed that the PSA level was an independent predictive factor for bRFS (*P* = 0.022) without any significant correlations with other probable risk factors such as GS, T-stage and PSM in the present study (Table 5), and the results are supported by other studies [8, 11, 13, 25, 26, 28].

In our study, BR was also frequently observed in patients with a short PSA-DT, although the PSA-DT was significantly correlated with other known risk factors (Table 6). In particular, 11 (85%) of 13 patients with a PSA-DT < 10 months compared with 1 (17%) of 6 with a PSA-DT ≥10 months developed a second BR event within 6 months after HS-RT (median: 3 vs 25 months). Furthermore, our results suggest that a reduction in the PSA level at 6 months after HS-RT can predict BR. Interestingly, these results may also suggest that patients with a long PSA-DT do not require frequent short-interval follow-up, if the reduction in the PSA level has remained >50% at the first evaluation at 3–6 months after S-RT, although long-term follow-up is necessary. On the other hand, patients with a PSA-DT < 10 months at the initiation of S-RT probably need careful follow-up every 2–3 months, especially during the first year after S-RT.

In the present study, the bRFS rates of patients with a high PSA level or short PSA-DT before HS-RT, as well as other risk factors, were also disappointing (Table 4), and a different approach such as combination ADT with S-RT may be necessary for these high-risk patients [29]. A randomized control trial (RTOG 9601) showed that a combination of 24-month antiandrogen therapy with S-RT improved not only bRFS but also OS in PC patients with BR after RP [30]. Similar to RTOG 9601, another randomized trial (GETUG-AFU 16) showed that an addition of goserelin for 6 months also improved progression-free survival after S-RT [31]. However, an appropriate candidate for ADT and S-RT combination therapy and the best timing and duration of ADT are still unknown.

The present study has several limitations. First, it was retrospective and the number of patients was small. Existence of confounding among the predictive factors determined by the multivariate analysis seems to be undeniable, although results obtained in the present study were consistent with those determined in previous studies [8, 11, 13, 25, 26, 28]. Second, the follow-up interval in the present study may be considered relatively short (median: 62 months). However, the PSA level in only three patients without BR exceeded 0.1 ng/ml at the last follow-up, but all of them survived without BR for more than 4 years after S-RT (Fig. 4). Lastly, six (16%) and eleven (29%) patients did not undergo pelvic MRI and bone scintigraphy as a whole body pre-HS-RT survey. Therefore, it was possible that some patients with bone metastases at the start of HS-RT were enrolled in the present study. In fact, 6 (55%) of 11 patients without examination of bone scintigraphy had further biochemical recurrences at 2–65 months (median: 18 months) after HS-RT, but there have been no clinical recurrences so far. The prospective study setting definite eligibility criteria for pre-screening will clarify the usefulness of PSA kinetics for predicting the risk of recurrence in S-RT for BR after RP.

In conclusion, our dose fractionation schedule for HS-RT in PC patients with BR after RP seems feasible. Despite the small number of patients in the present study, evaluation of PSA kinetics before and after HS-RT may be a useful tool to predict the risk of further recurrence and to determine the follow-up schedule for each patient. Accumulating data using S-RT alone will help us determine an appropriate combination therapy of S-RT plus ADT in the near future.

## CONFLICT OF INTEREST

None declared.
